# ITO film stack engineering for low-loss silicon optical modulators

**DOI:** 10.1038/s41598-022-09973-5

**Published:** 2022-04-15

**Authors:** Evgeniy S. Lotkov, Aleksandr S. Baburin, Ilya A. Ryzhikov, Olga S. Sorokina, Anton I. Ivanov, Alexander V. Zverev, Vitaly V. Ryzhkov, Igor V. Bykov, Alexander V. Baryshev, Yuri V. Panfilov, Ilya A. Rodionov

**Affiliations:** 1 FMN Laboratory, Bauman Moscow State Technical University, Moscow, 105005 Russia; 2 Dukhov Automatics Research Institute, (VNIIA), Moscow, 127055 Russia; 3 Institute for Theoretical and Applied Electromagnetics RAS, Moscow, 125412 Russia

**Keywords:** Electrical and electronic engineering, Materials for devices, Materials for optics, Nanoscale materials, Optics and photonics, Applied optics, Optical materials and structures, Optical physics, Nanoscale devices, Materials science

## Abstract

The Indium Tin Oxide (ITO) platform is one of the promising solutions for state-of-the-art integrated optical modulators towards low-loss silicon photonics applications. One of the key challenges on this way is to optimize ITO-based thin films stacks for electro-optic modulators with both high extinction ratio and low insertion loss. In this paper we demonstrate the e-beam evaporation technology of 20 nm-thick ITO films with low extinction coefficient of 0.14 (N_c_ = 3.7·10^20^ cm^−3^) at 1550 nm wavelength and wide range of carrier concentrations (from 1 to 10 × 10^20^ cm^−3^). We investigate ITO films with amorphous, heterogeneously crystalline, homogeneously crystalline with hidden coarse grains and pronounced coarsely crystalline structure to achieve the desired optical and electrical parameters. Here we report the mechanism of oxygen migration in ITO film crystallization based on observed morphological features under low-energy growth conditions. Finally, we experimentally compare the current–voltage and optical characteristics of three electro-optic active elements based on ITO film stacks and reach strong ITO dielectric permittivity variation induced by charge accumulation/depletion (Δn = 0.199, Δk = 0.240 at λ = 1550 nm under ± 16 V). Our simulations and experimental results demonstrate the unique potential to create integrated GHz-range electro-optical modulators with sub-dB losses.

## Introduction

There are several promising fields of silicon photonics like quantum computing, LIDARs, high-performance datacenters, neuromorphic computing and precision optical spectroscopy^1–5^, which can extremely benefit from low loss integrated optical modulators. A number of optical modulator implementations based on various physical effects have been already demonstrated (Table 1). The devices performance is typically defined by high speed, low loss, small footprint, fabrication flexibility and integration capabilities. One of the most commonly used modulator types is a lithium niobate modulator (Table 1A) based on the Pockels effect^6,7^. It exhibits low propagation loss (less than 0.1 dB) and a high modulation rate (over 40 GHz). The key remaining issue of lithium niobate modulators is the excessive active element length, which can exceed 2 mm. The next optical modulators type (Table 1B) is based on the free-carrier plasma dispersion effect (FCPD), where the active element is a p-n or p-i-n junction. Such modulators are based on charge carrier concentrations change^8,9^, they demonstrate high speed (over 30 GHz) and medium footprint area (active element length is 100–2000 μm). However, their insertion loss is significantly higher than that of lithium niobate modulators. Organic electro-optical materials (Table 1C) are also used to create high-performance devices based on the Pockels effect^10,11^. They provide both high speed (over 40 GHz) and small footprint (active element length down to 5 μm), but suffer from high propagation loss (about 2.5 dB in the active part). Another type of optical modulators, based on the Franz-Keldysh effect and the quantum-confined Stark effect (Table 1D), is implemented mainly in InGaAsP/InP or GeSi structures^12^. These modulators exhibit high speed (over 40 GHz), low loss (about 1 dB in the active part) and medium footprint. However, they require high fabrication and integration complexity. In the last 10 years, optical modulators based on a change in the dielectric constant of transparent conducting oxides (TCO), such as indium tin oxide (ITO)^13–16^, have attracted great interest (Table 1E). The accumulation (depletion) of free carriers in ITO provides a small footprint (active element length of about 5 μm) and low propagation loss (up to 0.5 dB). Their modulation frequency has been experimentally confirmed up to 1 GHz^16^, but higher rates can be achieved^17^. TCO-based devices demonstrate the trade-off between low propagation loss, small footprint and high modulation frequency, making them one of the most promising solutions. Moreover, all materials used in these devices are well compatible with planar silicon technology, making the full power of mature CMOS technology applicable to large-scale production of TCO optical modulators.

**Table 1:** Comparison of electro-optical modulators implementations.

Modulator type		Device active area length, um	Switch voltage, V	Extinction ratio, dB	3 dB bandwidth, GHz	Insertion loss (in the active area of a modulation), dB	References
A	Lithium niobate based (MZM or MRR)	150–5000	2.3–9	< 20	> 40	0.1–4	^6,7^
B	p-i-n and p-n junction based (MZM or MRR)	100–2000	1.8–6.5	3.1–7.5	> 30	1.5–6	^8,9^
C	NLM based (MZM or MRR)	4–1500	3.3–4	4–5	> 40	2.5–8	^10,11^
D	InGaAsP/InP based (MZM, EAM)	300–500	1.5–3	< 25	> 40	< 1	^12^
E	TCO based (MZM or MRR, EAM)	1.4–32	4–16	2.1–5	> 1	1–6	^13,15–17^

One of the most widely used TCO materials is indium tin oxide. Its dielectric constant depends on the carrier concentration and is described by the Drude–Lorentz model^18^, thus, it is possible to adjust both the real and the imaginary parts of the thin film refractive index^19^. This makes ITO a reasonable choice for the implementation of the modulator active element. Optical modulators based on thin film optical properties change effect could be realized as a hybrid waveguide, which represents a metal–oxide–semiconductor (MOS) capacitor with a built-in thin ITO layer. When an electrical potential is applied to active element, the charge accumulation layer is formed on the oxide/ITO interface where carrier concentration is significantly higher. The light mode localized in this sub-um volume is absorbed^13,14^ or changes the phase^15,16^. One of the key challenges for high-performance optical modulators is to achieve the required ITO thin film carrier concentration, which strongly affects the propagation loss and modulation depth of the thin film. ITO thin films with controllable carrier concentration are usually deposited with high-energy PVD methods such as ion beam deposition^15,16^, magnetron sputtering^20,21^ and pulsed laser deposition^22^ to avoid the following film annealing, which leads to strong carrier concentration increase. Ion-beam assisted deposition without subsequent annealing^23,24^ can also be a good choice for this purpose as it provides very good process control, but morphology and extinction coefficient of such thin films have not been investigated for ultrathin layers.

In this paper, we propose ITO thin films ion-beam assisted e-beam evaporation (IBAE) technology with subsequent annealing which provides versatile and accurate control of carrier concentration. Opposed to sputtering and ion-beam deposition the proposed IBAE process is characterized by lower energies avoiding intensive crystallization and formation of transition layer^25^. Electron beam evaporation is a very flexible technique; it allows deposition in ultra-high vacuum conditions with quite a small amount of extremely pure materials allowing ion assistance during process. Subsequent annealing in an inert atmosphere^26^ makes it possible to further improve the polycrystalline structure and properties of the film without a significant increase in the carrier concentration. In order to experimentally quantify the electro-optical effect, we fabricated and compared MOS capacitors based on the evaporated ITO films, which are similar to active elements of electro-optical modulators. The current–voltage characteristics of the capacitors and electro-optical effect in the accumulation/depletion layers were carefully measured and analyzed using spectroscopic ellipsometry^22,27,28^ under electrical biasing. More than 80 samples were experimentally measured to determine the materials properties and films stack optimization.

## Results and discussion

### ITO thin films investigation

The key technology for modern ITO-based optical modulators is thin ITO films with a strong compromise between carrier concentration and optical properties. Using our optical modulator simulations (see Supplementary) we found that both minimal device length and propagation loss can be achieved with the ITO films carrier concentrations in the range of 0.5 to 2 × 10^20^ cm^−3^. With the typical value of the carrier mobility in ITO films equals to 15 cm^2^/(V s)^18,29^ we calculated the resistivity (ρ = 1/μ_c_N_c_e), which should be in the range from 2 to 8 × 10^–3^ Ω cm. This range will be used to estimate resistivity values ​​throughout the work.

**Figure 1:**
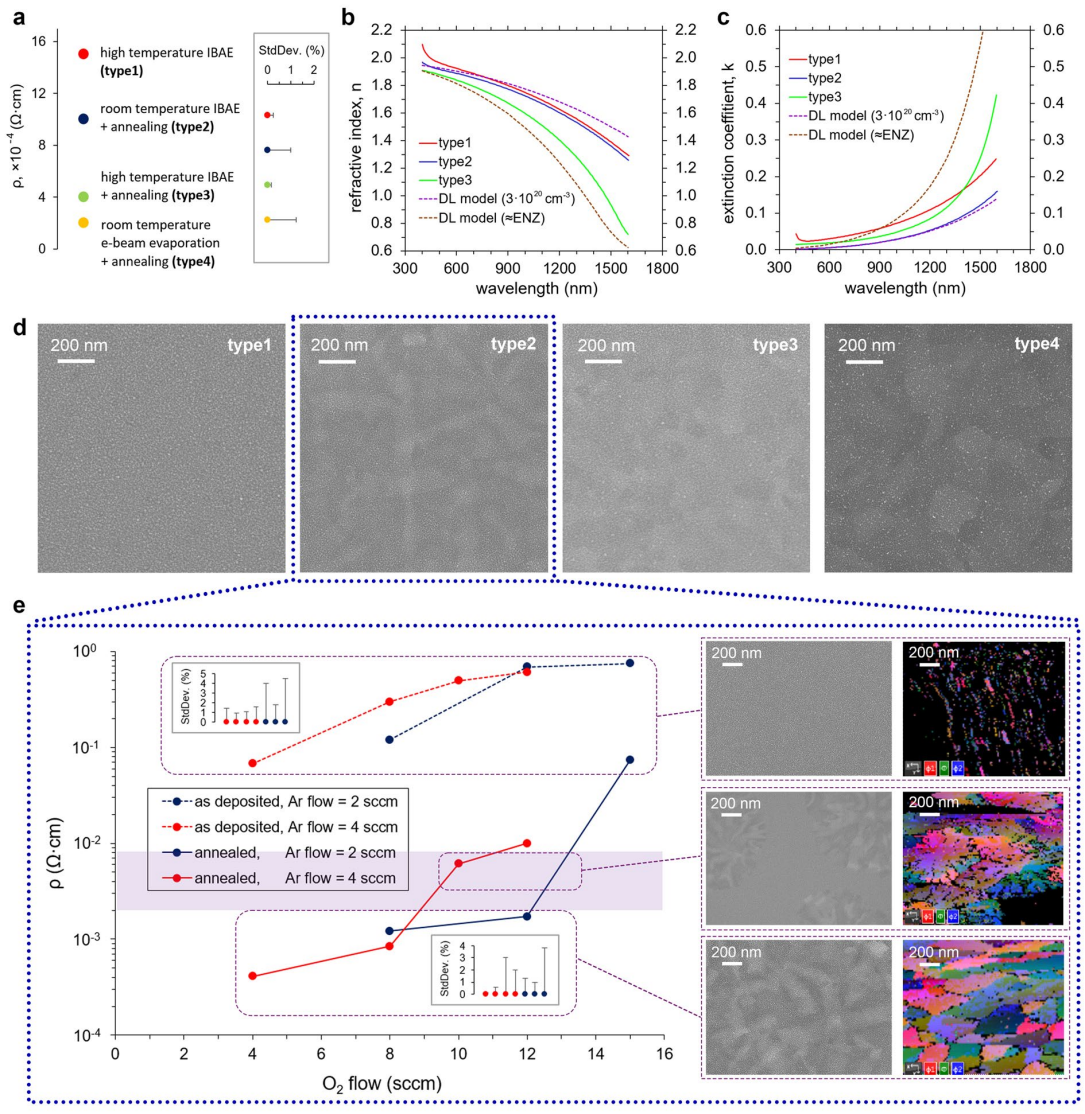
(**a**) ITO films resistivity distribution for proposed four deposition technique; ITO films (**b**) refractive index and (**c**) extinction coefficient dependences on wavelength; (**d**) High-resolution SEM images of the ITO films surface; (**e**) Room temperature IBAE + annealing ITO films resistivity dependence on Ar/O_2_ mixture composition during evaporation. Resistivity standard deviation error bars are shown for each point of the graphs from left to right correspondingly. Selected areas show the surface (SEM images) and crystallography (SEM EBSD maps) of films with different resistivity values.

We first compared the resistivity, optical properties, and surface morphology (Fig. 1) of the 20 nm-thick ITO films evaporated with four different techniques: (1) high temperature IBAE (type1); (2) room temperature IBAE with the subsequent 300 °C annealing (type2); (3) high temperature IBAE with the subsequent 300 °C annealing (type3); (4) room temperature e-beam evaporation with the subsequent 300 °C annealing (type4). We observe strong variation in films resistivity measured by the four-probe method with respect to evaporation technology and annealing parameters (Fig. 1a, for a detailed description see the Supplementary). The resistivity of the ITO film deposited at room temperature without annealing (not shown) is more than 10^–1^ Ω cm, which indicates insufficient crystallization of the film and a large amount of nonactivated interstitial oxygen^30–32^. All the evaporated ITO films are ultra-flat with the film surface RMS roughness of 0.7 ± 0.2 nm. By analyzing high quality SEM images of films one can observe a strong texture growth for the annealed films (Fig. 1d, type 2–4) and homogeneous microstructure for the film evaporated under elevated temperature without subsequent annealing.

The optical constants of films (type 1–3) were measured in the wavelength range from 400 to 1600 nm (Fig. 1b,c). Film type4 was not investigated, because its carrier concentration was too high (estimated carrier concentration from the resistivity measurement was about 8.3 × 10^20^ cm^−3^, which is above the epsilon-near-zero point (ENZ)—about 6.5 × 10^20^ cm^−3^ for λ = 1550 nm, this value will lead to extremely high losses in device). Obviously, film type1 has a much higher extinction coefficient in the entire visible range (Fig. 1c), which can be explained by low crystallization of the film during evaporation. The strong texture of the film type2 indicates an intense crystallization process that results in the lowest absorption over the entire wavelength range. ITO film type3 also has a strong crystalline structure (Fig. 1d) and a relatively low extinction coefficient in the visible region. Carrier concentrations for ITO films (type 1–3) are calculated based on the measured plasma frequency ω_p_ from ellipsometry data (see Supplementary) to be 4.2 × 10^20^ cm^−3^ (type1), 3.7 × 10^20^ cm^−3^ (type2), 5.7 × 10^20^ cm^−3^ (type3). The carrier mobilities for films (type 1–3) are calculated as μ = 1/ρeN_c_ and equal 9.4 cm^2^/V s (type1), 15.5 cm^2^/V s (type2), 27.0 cm^2^/V s (type3). According to previously published data^18^, ITO films (type1 and 2) are promising candidates to be successfully used in electro-optical modulators in both n-dominant (Mach–Zehnder) and k-dominant (electro-absorption) regions. The value for film type3 is closest to the ENZ point at λ = 1550 nm (Fig. 1b,c, Drude–Lorenz model for ENZ), therefore, it can be used for electro-optical elements that require switching the dielectric constant through the ENZ point^33,34^.

However, low-loss n-dominant electro-optic modulator requires ITO films with higher resistance values (from 2 × 10^–3^ to 8 × 10^–3^ Ω cm). One can clearly see that the resistance of ITO films is directly related to their crystalline structure and morphology. Two methods of controlling the resistance of ITO films have been studied: (1) annealing at different temperatures; (2) Ar/O_2_ gas mixture variation during IBAE with subsequent annealing. Due to the minimal extinction coefficient and close to the desired resistivity, we chose the film type2 deposition recipe for further investigation. We studied ion-beam assisted e-beam evaporation of 20 nm-thick ITO films at room temperature with the various Ar/O_2_ mixture ratio (Ar flow of 2 and 4 sccm, O_2_ flow of 4, 8, 10, 12, 15 sccm) without and with subsequent 20-min annealing in argon atmosphere at 300 °C (Fig. 1e). One can see that annealing of the films makes it possible to reduce their resistance close to the desired region in a controlled manner. ITO films with a relatively high resistance (more than 10^–2^ Ω cm) have amorphous or nanocrystalline structure (Fig. 1e, no crystalline phases were found in the SEM EBSD analysis). Films with relatively low resistivity (less than 10^–3^ Ω cm) have direct evidence of forming crystalline structure (grains with hidden boundaries were detected by SEM EBSD analysis). Unfortunately, the film (Fig. 1e, Ar flow of 4 sccm, O_2_ flow of 10 sccm) with the resistance from the desired region from 2 to 8 × 10^–3^ Ω cm (corresponds to the required range of charge carrier concentrations) have a crystalline structure with inclusions amorphous phase. This is a stoichiometrically heterogeneous structure that can lead to an uneven carrier distribution in optical modulator devices.

**Figure 2:**
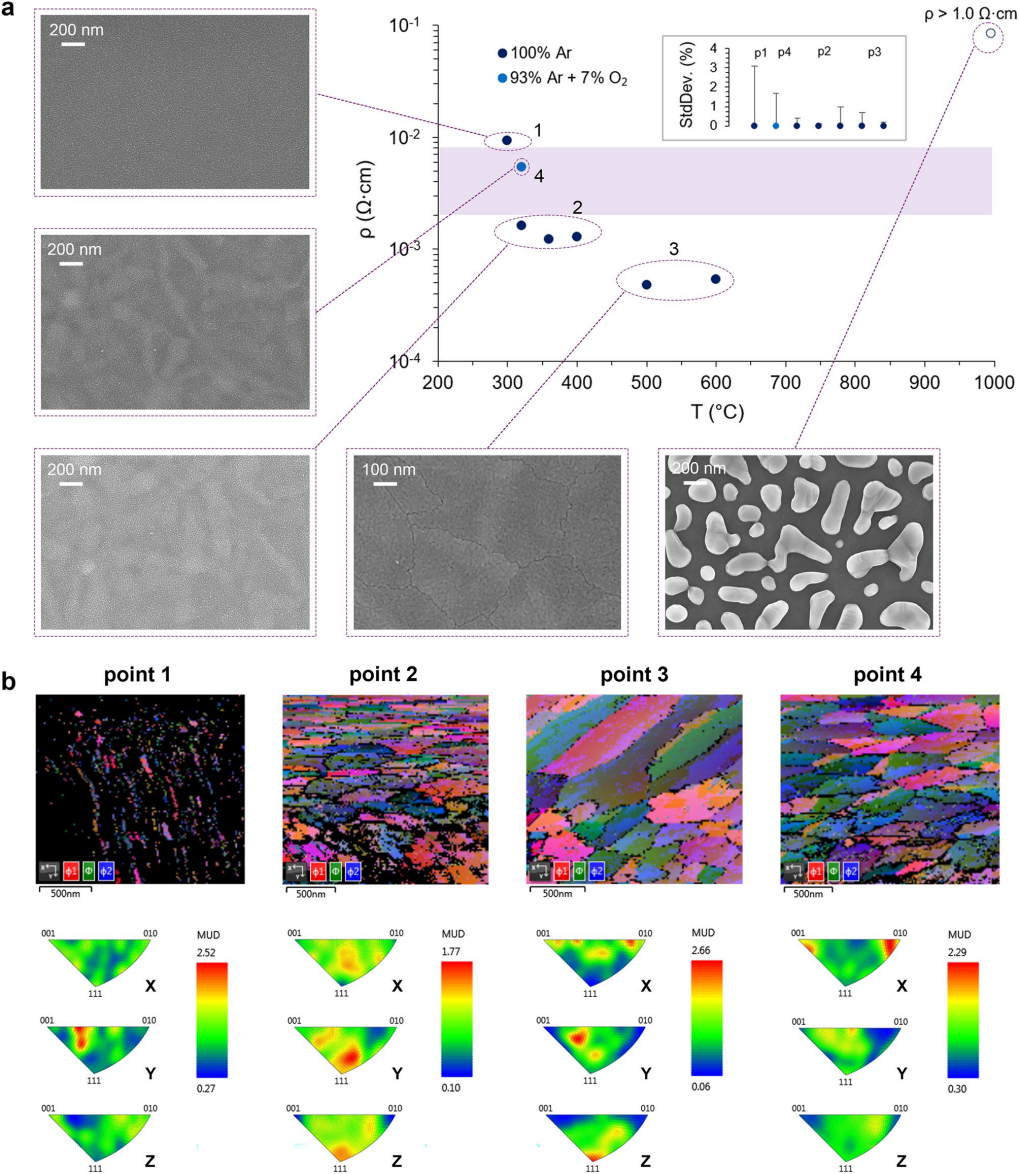
(**a**) 20 nm-thick ITO films [IBAE at room temperature in an Ar/O_2_ mixture (4/12 sccm)] resistivity dependency on annealing temperature and atmosphere; high-resolution SEM images of the ITO films surface for each annealing parameters region; resistivity standard deviation error bars are shown for each point of the graphs from left to right correspondingly. (**b**) SEM EBSD analysis for the typical ITO films corresponding to each annealing parameters region.

In order to achieve much wider process window and get electrical, optical, and morphological properties of ITO films simultaneously, we investigated the dependence of the resistance and the crystalline structure on annealing parameters (Fig. 2a). We chose the closest resistivity value to the desired range which corresponds to IBAE at room temperature in an Ar/O_2_ mixture (Ar flow = 4 sccm and O_2_ flow = 12 sccm) and varied annealing temperature and atmosphere. One can notice that the ITO film annealed at 300 °C remains amorphous (Fig. 2a, point 1), while increasing the temperature by only 20 °C immediately initiates crystallization (Fig. 2a, point 2). We marked stepwise crystallization behavior, which results in a well-defined ITO crystalline structure when the annealing temperature rises above 500 °C (Fig. 2a, point 3). Under higher temperatures (in the range from 600 to 1000 °C) films damage, melt and break into islands by surface tension forces. It should be noted that annealing in the atmosphere with a small fraction of oxygen increases resistance significantly (Fig. 2a, point 4). In this case, films have a crystalline structure, in contrast to partial crystallization (Fig. 1e), which is observed for the films with a resistivity in the range from 2 to 8 × 10^–3^ Ω cm, which were annealed in an atmosphere of pure argon. All resistivity values ​correlate with carrier concentration values obtained from the ellipsometry data with Tauc–Lorenz model for each ITO film in the UV range (see Supplementary). SEM EBSD analysis (Fig. 2b) demonstrates that raising the annealing temperature in an argon atmosphere (Fig. 2b, points 1–3) leads to increasing the pole density of reflecting planes orientations of ITO films (see inverse pole figures—IPF). This indicates the appearance of preferred grains orientations in ITO films. It should be noticed, that the film annealed in the oxygen-diluted atmosphere (point 4) had another grains orientation.

**Figure 3:**
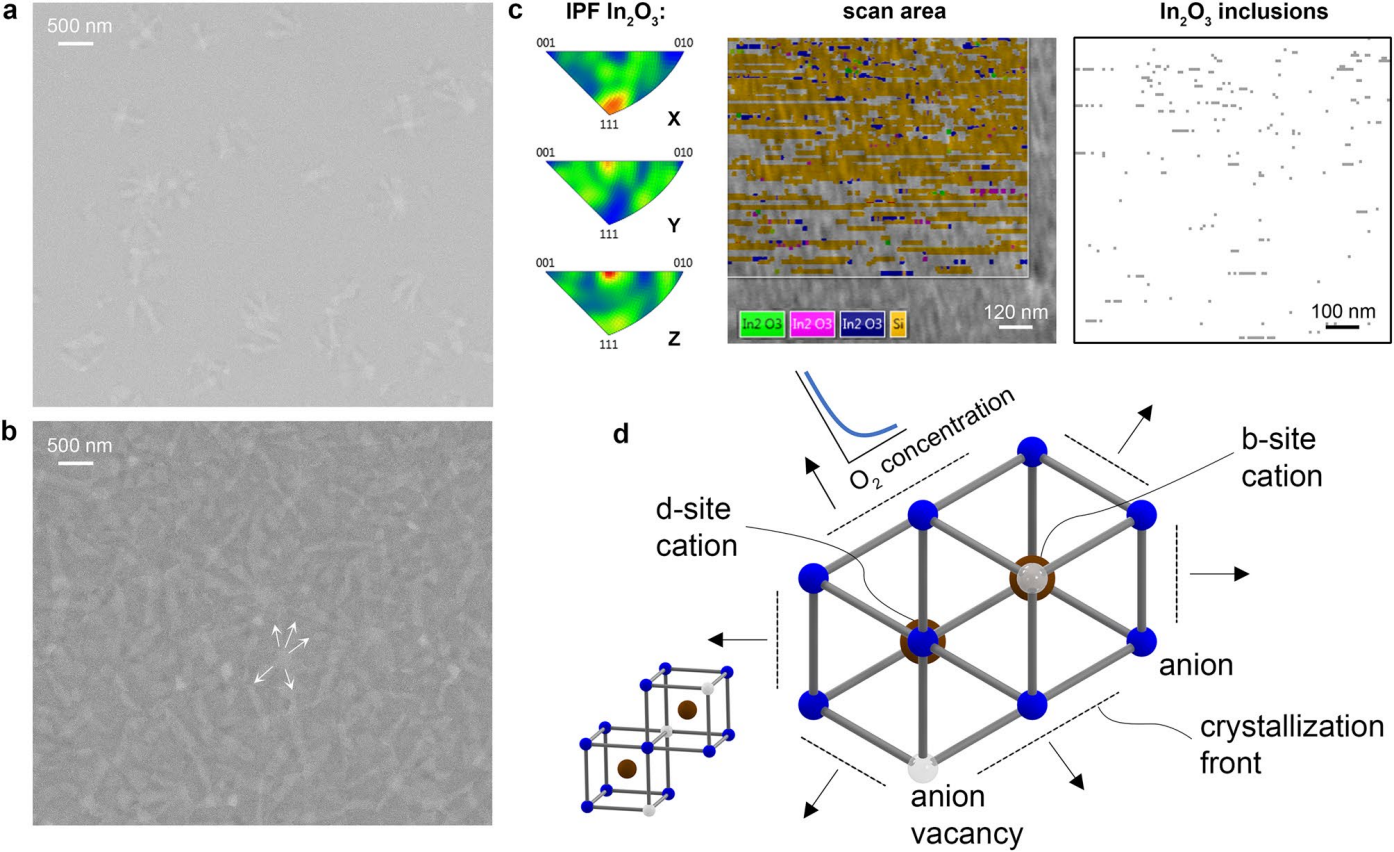
20 nm-thick ITO films (room temperature IBAE in an Ar/O_2_ mixture 2/10 sccm) with different annealing temperatures: (**a**) 300 °C—crystalline structure with inclusions of amorphous phases; (**b**) 320 °C—crystalline structure; (**c**) SEM EBSD analysis of an non-annealed ITO film (room temperature IBAE in an Ar/O_2_ mixture 2/10 sccm), one can see nucleus of crystalline In_2_O_3_ phase; (**d**) Projection of the ITO lattice on < 222 > plane and the crystallization process (oxygen diffusion along the direction of the crystallization front).

One can notice that IBAE films have structural crystalline features and represent chemically heterogeneous systems prior to annealing. Since the energy in the IBAE processes is higher, the formation of crystal nuclei occurs during the evaporation process (which is confirmed by the EBSD analysis for the non-annealed thin film—Fig. 3c). Based on the SEM image analysis (Fig. 3a,b), we assume that during annealing the propagation of the crystallization front from the nucleus along the growth channels occurs in preferred orientations similar to processes in liquid-phase epitaxy^35^. In this case, the movement of the crystallization front can form the opposite diffusion flow which is perpendicular to the growing surface (Fig. 3d). We suggest that during ITO crystallization a depletion zone appears where oxygen diffuses to form a crystal lattice. SEM images (Fig. 3a,b) demonstrate from 2 to 6 channels extending radially from each central point, which may be oxygen diffusion channels.

We observed that annealing of ITO films deposited without ion assistance causes a greater drop in resistance (Fig. 1a). Upon an e-beam evaporation process some part of ITO thin film can crystallize (Fig. 3c). For ion-assisted processes, oxygen atoms and ions outside the crystal lattice are embedded into the volume of the film^30–32^. This affects further crystallization during annealing when oxygen is annihilated with vacancies^36^. Wherein the crystalline phase is more localized in films e-beam evaporated with ion assistance. Thus, the proposed deposition technique utilizes lower energies and higher oxygen concentrations, which distinguishes it from methods described in recent papers^23,24^. These deposition conditions favor the formation of noncrystalline films, as evidenced by their low carrier concentration (all non-annealed ITO films in this work have N_c_ < 4 × 10^18^ cm^−3^ when deposited at a room temperature). The films deposited at a higher energy and lower oxygen concentration without further annealing show higher carrier concentrations (N_c_ > 1 × 10^19^ cm^−3^) and higher degree of crystallization. In this case oxygen has enough time and energy to integrate into the crystal lattice vacancies and initiate crystallization process, whereas in the proposed technique it appears in the form of embedded oxygen outside the crystal lattice. The annealing process under controlled temperature and ambient provides the certain amount of energy to activate oxygen, which guarantee precise regulation of the carrier concentration: N_c_ in the range from 1 to 10 × 10^20^ cm^−3^.

### Electro-optical modulation

**Figure 4:**
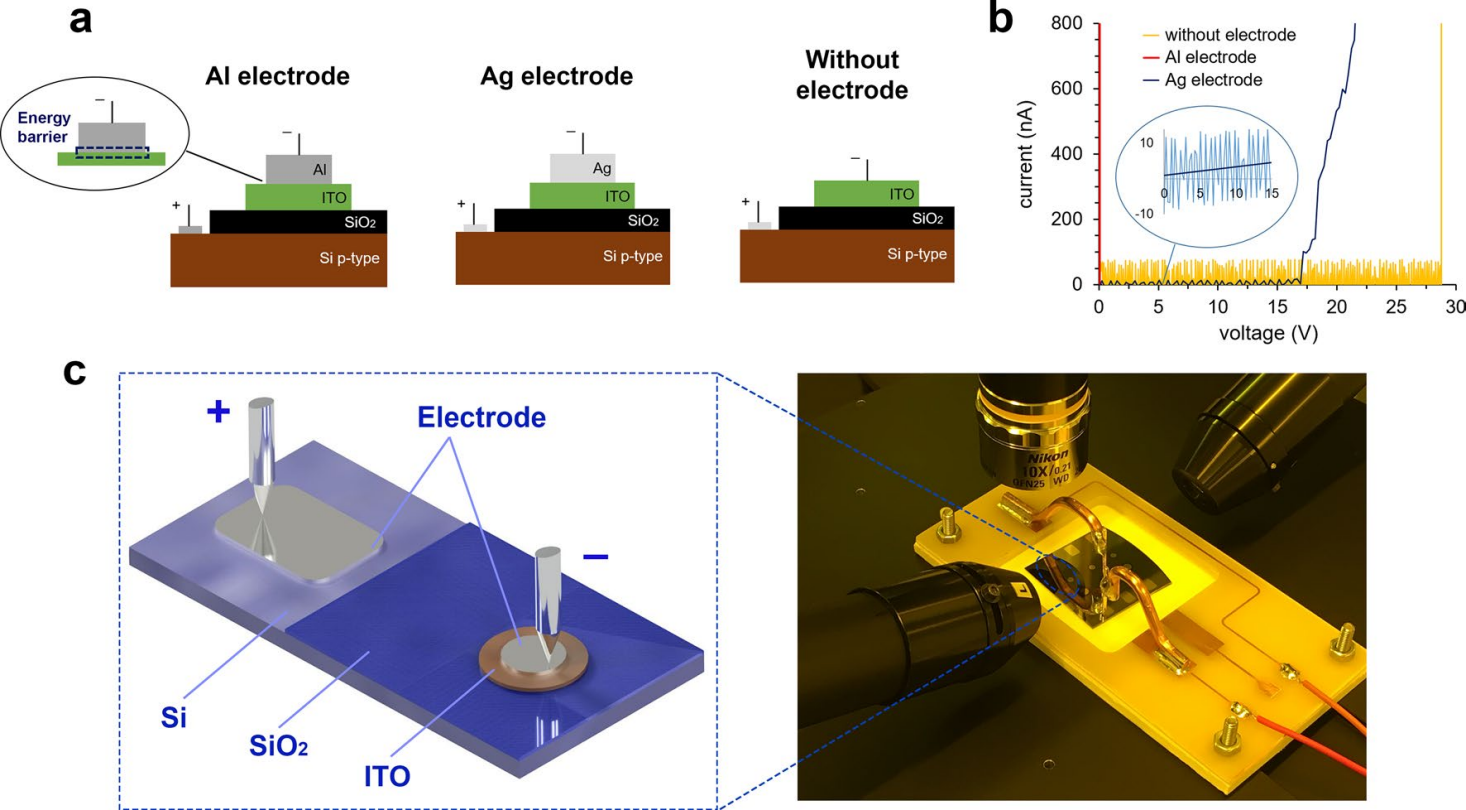
(**a**) Three ITO-based films stacks (MOS capacitors) for electrical characterization (the breakdown zone is shown with dotted area); (**b**) current–voltage characteristics of the fabricated MOS capacitors; (**c**) 3D-model and photograph of the experimental setup.

We fabricated MOS capacitors (Si/SiO_2_/ITO/Me, Fig. 4a) based on ITO films (type 1–3) using aluminum (Al, work function 4.2 eV) and silver (Ag, 4.8 eV) electrodes (see “Methods”). MOS capacitors with the aluminum electrodes exhibit breakdown voltages of less than 20 mV (Fig. 4b, red vertical curve). When replacing aluminum electrode with the silver one, a significantly higher breakdown voltage of 17 V was observed at the leakage current of less than 4 nA (Fig. 4b, blue curve). Additionally, we tested the structure (Si/SiO_2_/ITO) without metal electrode (the probes were placed in direct contact with ITO film) that showed biggest breakdown voltage of 29 V (Fig. 4b, yellow curve). High noise level in this case can be explained by poor contact between the probe and ITO film surface. Based on these measurements we assumed that Al/ITO pair forms an energy barrier at their interface. When an electric potential is applied, electrons are emitted due to the lower Al work function compared to ITO (4.2 eV versus 4.7 eV, respectively, Fig. 4a—dotted area). This means that an energy barrier of 0.5 eV appears at the Al/ITO interface ($\Delta {\varphi }_{ms}={\Phi }_{ITO}-{\Phi }_{Al}$). The emission current density in this case is defined as:

1 $$j={j}_{ITO\to Al}-{j}_{Al\to ITO}=\frac{1}{4}q{n}_{S}{v}_{0}\left({e}^{\beta Vg}-1\right),$$

where q is an electron charge, n_s_ = n_0_e^–βΔφ^—surface concentration in a semiconductor/metal interface, β = q/kT, υ_0_—thermal velocity of electrons. In this case the free path of electrons in ITO equals to 17.8 nm:

2 $${l}_{e}=\frac{h}{\rho {q}^{2}N{\lambda }_{e}},$$

where h is the Plank’s constant, 1/λ_e_ = (3π^2^N)^1/3^.

**Figure 5:**
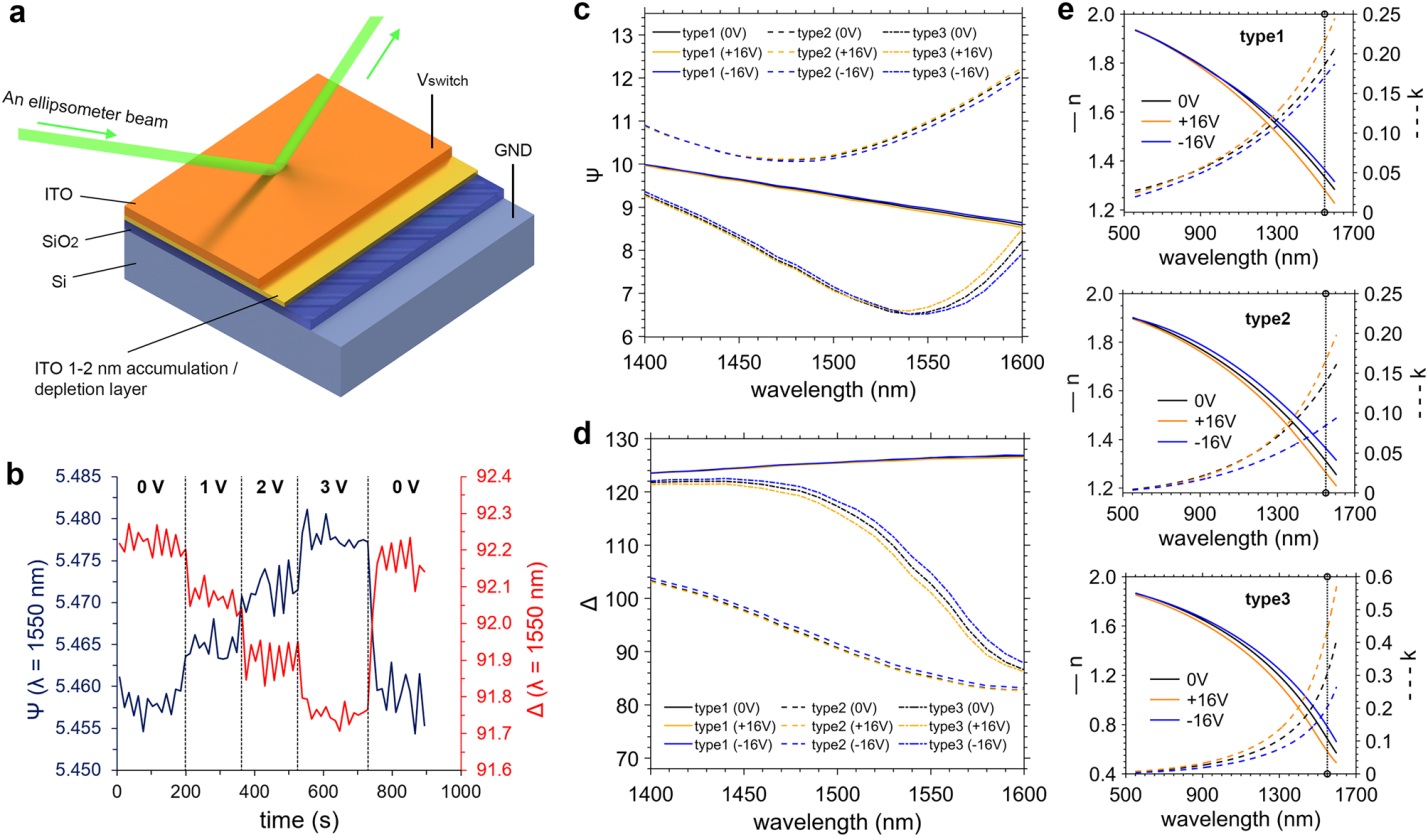
(**a**) Ellipsometry model measurement scheme; (**b**) ITO (type3) ψ and Δ at wavelength of 1550 nm; (**c**) ψ; (**d**) Δ of the multilayer stack in the wavelength range from 1400 to 1600 nm at voltages of − 16 V and + 16 V; (**e**) film types 1–3 accumulation layer n and k in the wavelength range from 550 to 1600 nm at voltages of 0, + 16 and − 16 V.

The calculated electric current in this barrier for the chosen electrode area (about 2 mm^2^) is about 0.19 A (at applied voltage of 20 mV—Eq. 1). Additionally, free electrons can be accelerated by external electric field and pass through ITO layer (electron mean free path is comparable to the film thickness), which can lead to oxide destruction and breakdown. Next, we investigated ITO films optical characteristics switching under applied voltage (for detailed information see Supplementary). First, the structure (Si/SiO_2_/ITO) without electrode was used in order to achieve better ellipsometry sensitivity (due to the lack of a semitransparent layer) and a higher breakdown voltage (Fig. 5a). Then, ellipsometry parameters psi and delta (ψ, Δ) were carefully measured in the wavelength range from 400 to 1600 nm for multilayer stacks with ITO films (type 1–3). We observed stronger effect of the applied voltage on Psi and delta at longer wavelengths in the range from 1400 to 1600 nm (Fig. 5c,d). The dependences of the applied voltage (the data was validated only from 0 to + 3 V for the ITO film type3) on the ellipsometry parameters ψ and Δ were measured at a standard telecommunication wavelength of 1550 nm (Fig. 5b).

The optical parameters of the ITO thin films were carefully investigated based on ellipsometry measurements (see Supplementary). The complex refractive index of the accumulation layer was calculated using the Drude–Lorentz model in the wavelength range from 550 to 1600 nm (Fig. 5e). Note, that we adopted a homogeneous uniform accumulation layer approximation in the ellipsometry model (Fig. 5a) that satisfied the required level of detail. One can see, that the effect on the ellipsometry parameters of the film type1 turns out to be lower than that of the film type2, despite higher carrier concentration. Presumably, this can be explained by the presence of electron traps arises from the less crystallized structure of the film type1, which prevents the accumulation of charge. The film type3 has the strongest electro-optical effect due to the highest carrier concentration. We compared Δn and Δk for the ITO films (type 1–3) at 1550 nm (Table 2), the refractive index change under applied voltage for them is lower than indicated in recent works (Table 3). We assume that this associated with the large area of the millimeter-size electrode we used, which results in weaker accumulation (depletion) in the ellipsometer spot region due to unevenly distributed probe charge.

**Table 2:** Comparison of refractive index and extinction coefficient change for the various ITO films at λ = 1550 nm.

Parameter	type1	type2	type3
N_c_, cm^−3^	4.2 × 10^20^	3.7 × 10^20^	5.7 × 10^20^
Δn (0 + 16 V)	0.053	0.048	0.101
Δn (0–16 V)	0.030	0.053	0.098
Δk (0 + 16 V)	0.030	0.028	0.119
Δk (0–16 V)	0.016	0.056	0.121
Δn	0.083	0.101	0.199
Δk	0.046	0.084	0.240
n (at 0 V)	1.342	1.308	0.675
k (at 0 V)	0.226	0.141	0.329

**Table 3:** Comparison of ITO characteristics and electro-optical device parameters in recent works.

Device parameters			ITO characteristics						References
IL, dB	L_device_, um	V_switch_, V	t_ITO_, nm	N_c_, cm^-3^	n_ITO_ (1550 nm)	k_ITO_ (1550 nm)	Δn_ITO_ (1550 nm)	Δk_ITO_ (1550 nm)	
**Electroabsorption design**									
1	5	± 5	10	1.1 × 10^19^	1.964 (1310 nm)	0.002 (1310 nm)	0.922 (1310 nm)	0.271 (1310 nm)	^13^
–	1700	− 5	50	1.87 × 10^20^	1.75	0.195	0.08	0.075	^23^
**Mach–Zehnder design**									
6	32	± 6	10	2.29 × 10^20^	1.45	0.18	~ 1	–	^15^
–	2	± 16	10	2.3 × 10^20^	1.62	0.15	0.15	0.1	^37^
6	1.5	± 13	10	3.13 × 10^20^	1.44	0.12	0.44	0.11	^16^
**MOS-stack ellipsometry investigation**									
–	–	+ 5	40	6.62 × 10^20^	0.63	0.57	0.061	0.079	^22^
–	–	± 16	20	5.70 × 10^20^	0.81	0.33	0.199	0.240	Our work
–	–	± 16	20	3.79 × 10^20^	1.31	0.14	0.101	0.084	Our work

Finally, we calculated (see Supplementary), that such device implementation can potentially provide an electro-optical modulation frequency of approximately 3.4 GHz (with silver electrodes placed on top of the ITO layer). In further work, we suppose device implementation with single-crystalline Ag electrode^38,39^, which could abate losses induced by SPP propagation at electrode interfaces. Moreover, there are ITO MOS capacitor designs based on the tunable guided-mode resonance effect, which can greatly increase the Δn value^40^.

## Summary and conclusions

In conclusion, we developed ITO thin films and MOS capacitors deposition technique using ion-beam assisted e-beam evaporation with subsequent annealing for low-loss electro-optical modulation devices. With the proposed technique we deposited ultra-flat (RMS surface roughness from 0.6 to 0.8 nm) 20 nm-thick ITO films with the low carrier concentration up to 1 × 10^20^ cm^−3^. A direct dependence of ITO thin films resistivity on their crystal structure has been demonstrated. SEM film surface and SEM EBSD analysis allowed us to propose a mechanism of putative oxygen diffusion channels arising during films crystallization deposited using ion-beam assisted e-beam evaporation, which is the result of growing In_2_O_3_ nucleus crystalline phase during deposition. To experimentally quantify an electro-optical modulation effect we designed, fabricated and tested MOS structures based on ITO thin films stacks (with Al, Ag and without electrode) with high breakdown voltages of 17 to 29 V. A model that explains the experimental breakdown voltages for the different types of electrodes of the MOS capacitors is proposed and experimentally evaluated. The MOS structure with the silver electrode demonstrated significantly higher breakdown voltage compared to the aluminum one. Finally, we observed a strong electro-optical modulation of the complex refractive index (Δn = 0.199, Δk = 0.240) at a wavelength of 1550 nm in a voltage range from + 16 to − 16 V. It is worth noticing, that ITO films deposited at a higher temperature without subsequent annealing had the worst electro-optical the effect. These results demonstrate the possibility to create integrated electro-optical modulators in the GHz range with low propagation losses (theoretically estimated losses are less than 4 dB without device design optimization).

## Methods

### ITO deposition

ITO thin films were deposited on prime-grade doped Si < 100 > substrates (10–20 Ω cm) using a 10 kW electron beam evaporator (Angstrom Engineering) at a base pressure below 3 × 10^–8^ Torr. We first cleaned the substrates in a 2:1 solution of sulfuric acid and hydrogen peroxide (80 °C) and then rinsed with isopropanol to remove organic matter. We then immersed the plates in 49% hydrofluoric acid for about 20 s to remove the natural oxide layer. All films were grown using pieces of ITO with a 99.999% purity (In_2_O_3_—90%, SnO_2_—10%). The films were deposited at a rate of 2 Å/s, measured with a quartz monitor at an approximate distance from the source to the substrate of 30 cm. The thickness of all ITO thin films was 20 ± 2 nm. Ion assistance (accelerating voltage 70 V) and deposition on a substrate with an elevated temperature (150 °C) were performed to study their effect on the structure of the thin film, resistivity, and carrier concentration. Subsequent annealing was carried out in an argon atmosphere for 20 min.

### Resistivity measurement

The resistivity was measured using a four-point probe method using a ResMap 178 (CDE) four-probe surface resistance measurement system. We used a soft probe with a large footprint (500-μm tip radius semispherical probe) and a low clamping force (70 g) to ensure defect-free contact for the deposited films. A series of at least 5 measurements for more than 80 samples showed the same stable linear current–voltage characteristics. SEM analysis shows no damaged ITO films after measurements with a four-point probe.

### Scanning electron microscopy

To check the quality and uniformity of the deposited layers, the surfaces of the ITO films were examined using a Zeiss Merlin scanning electron microscope with a Gemini II column immediately after deposition. All SEM images were obtained using an in-lens detector and an accelerating voltage of 5 kV at a working distance from the sample to the detector of 1 to 4 mm. Magnifications of 3 k, 7 k, 15 k and 50 k were used for complete analysis of the samples.

### Electron backscatter diffraction

The crystallinity of the obtained ITO thin films was investigated by electron backscattering diffraction (EBSD) using a Nordlys-II S electron backscattering detector (Oxford Instruments) at an accelerating voltage of 10 keV at a working distance of 12.5 mm and a tilt angle of the sample into the microscope chamber of 70°. The AZtec 3.3 software was used to analyze the area. In paper Quaas et al.^41^ XRD analysis of ITO films showed In and In_2_O_3_ phases, while Sn and SnO_x_ phases were not detected (it is assumed that the phases are embedded in the In/In_2_O_3_ lattice). Here, the analysis similarly used the In_2_O_3_ phases (lattice—cubic minimum; space group—206; diffraction symmetry class: m-3, a, b, c = 10.12 Å, Alpha—90°, Beta—90°, Gamma—90°). The search for the SnO_x_ phases gave no results, as expected.

### Stylus profilometry

The stylus profiler KLA Tencor P17 (with Durasharp 38-nm tip radius stylus) was used. All measurements were completed using 0.5 mg taping strength, scan rate 2 μm/s and 20 μm scanned line length.

### Active element layer stack fabrication

To fabricate a multilayer stack, the p-type doped Si < 100 > substrates (0.001 Ω cm) were oxidized for 6 min at 1000 °C forming 20 nm-thick silicon oxide layer. 20 nm-thick ITO films with an area from 2 to ​​5 mm^2^ were formed on oxidized silicon substrates by the deposition of shadow masks. Then, 100 nm-thick aluminum or silver electrodes were deposited at room temperature (deposition parameters can be found elsewhere^42^) on the ITO and Si surfaces (Fig. 4a) to ensure better contact during electrical tests. The sample was connected to the power supply unit using a homemade contact tool mounted on the table of the ellipsometer (Fig. 4c). For optical measurements, we used additional focusing on a microspot about 200 μm in size. Experimental benches based on Sentech SE 800 PV and Woollam V-VASE ellipsometers have been developed. It allows to measure the change in ellipsometric parameters ψ and Δ for MOS structures and the optical properties of thin films under the action of an applied voltage.

## Supplementary Information

Supplementary Information.

## References

[1] Poulton CV, Yaacobi A, Cole DB, Byrd MJ, Raval M, Vermeulen D, Watts MR (2017). Coherent solid-state LIDAR with silicon photonic optical phased arrays. Opt. Lett..

[2] Baburin AS, Ivanov AI, Rodionov IA (2018). Highly directional plasmonic nanolaser based on high-performance noble metal film photonic crystal. Nanophotonics VII..

[3] Cheng Q, Rumley S, Bahadori M, Bergman K (2018). Photonic switching in high performance datacenters. Opt. Express.

[4] Amin R, George JK, Sun S, Ferreira de Lima AN (2019). ITO-based electro-absorption modulator for photonic neural activation function. APL Mater..

[5] Shastri BJ, Tait AN, Ferreira de Lima T (2021). Photonics for artificial intelligence and neuromorphic computing. Nat. Photonics.

[6] Wang C, Zhang M, Chen X (2018). Integrated lithium niobate electro-optic modulators operating at CMOS-compatible voltages. Nature.

[7] Witmer J, Valery J, Arrangoiz-Arriola P (2017). High-Q photonic resonators and electro-optic coupling using silicon-on-lithium-niobate. Sci. Rep..

[8] Green WM, Rooks MJ, Sekaric L, Vlasov YA (2007). Ultra-compact, low RF power, 10 Gb/s silicon Mach–Zehnder modulator. Opt. Express.

[9] Xiao X, Xu H, Li X, Li Z, Chu T, Yu Y, Yu J (2013). High-speed, low-loss silicon Mach–Zehnder modulators with doping optimization. Opt. Express.

[10] Haffner C, Chelladurai D, Fedoryshyn Y (2018). Low-loss plasmon-assisted electro-optic modulator. Nature.

[11] Palmer R, Alloatti L, Korn D (2013). Low power Mach–Zehnder modulator in silicon-organic hybrid technology. IEEE Photonics Technol. Lett..

[12] Paul, K. L. *et al.* Design and fabrication of InGaAsP/InP waveguide modulators for microwave applications. In *Optical Technology for Microwave Applications VI and Optoelectronic Signal Processing for Phased-Array Antennas III*, vol. 1703 (International Society for Optics and Photonics, 1992). 10.1117/12.138398.

[13] Sorger VJ, Lanzillotti-Kimura ND, Ma RM, Zhang X (2012). Ultra-compact silicon nanophotonic modulator with broadband response. Nanophotonics.

[14] Li E, Gao Q, Chen RT, Wang AX (2018). Ultracompact silicon-conductive oxide nanocavity modulator with 0.02 lambda-cubic active volume. Nano Lett..

[15] Amin R, Maiti R, Carfano C (2018). 0.52 V mm ITO-based Mach–Zehnder modulator in silicon photonics. APL Photonics.

[16] Amin R, Maiti R, Gui Y (2021). Heterogeneously integrated ITO plasmonic Mach–Zehnder interferometric modulator on SOI. Sci. Rep..

[17] Tahersima MH, Ma Z, Gui Y (2019). Coupling-enhanced dual ITO layer electro-absorption modulator in silicon photonics. Nanophotonics.

[18] Cleary JW, Smith EM, Leedy KD, Grzybowski G, Guo J (2018). Optical and electrical properties of ultra-thin indium tin oxide nanofilms on silicon for infrared photonics. Opt. Mater. Express.

[19] Amin R, Suer C, Ma Z, Sarpkaya I, Khurgin JB, Agarwal R, Sorger VJ (2017). Active material, optical mode and cavity impact on nanoscale electro-optic modulation performance. Nanophotonics.

[20] Lee HW, Papadakis G, Burgos SP (2014). Nanoscale conducting oxide PlasMOStor. Nano Lett..

[21] Gui Y, Miscuglio M, Ma Z (2019). Towards integrated metatronics: A holistic approach on precise optical and electrical properties of Indium Tin Oxide. Sci. Rep..

[22] Xian S, Nie L, Qin J (2019). Effect of oxygen stoichiometry on the structure, optical and epsilon-near-zero properties of indium tin oxide films. Opt. Express.

[23] Rajput S, Kaushik V, Jain S (2019). Optical modulation in hybrid waveguide based on Si-ITO heterojunction. J. Lightwave Technol..

[24] Meng LJ, Teixeira V, Dos Santos MP (2010). Effect of the deposition rate on ITO thin film properties prepared by ion beam assisted deposition (IBAD) technique. Phys. Status Solidi (A).

[25] Sytchkova A, Zola D, Bailey LR, Mackenzie B, Proudfoot G, Tian M, Ulyashin A (2013). Depth dependent properties of ITO thin films grown by pulsed DC sputtering. Mater. Sci. Eng. B.

[26] Ali HM, Mohamed HA, Mohamed SH (2005). Enhancement of the optical and electrical properties of ITO thin films deposited by electron beam evaporation technique. Eur. Phys. J. Appl. Phys..

[27] Ma Z, Li Z, Liu K, Ye C, Sorger VJ (2015). Indium-tin-oxide for high-performance electro-optic modulation. Nanophotonics.

[28] Feigenbaum E, Diest K, Atwater HA (2010). Unity-order index change in transparent conducting oxides at visible frequencies. Nano Lett..

[29] Liu C, Matsutani T, Asanuma T, Kiuchi M (2003). Structural, electrical and optical properties of indium tin oxide films prepared by low-energy oxygen-ion-beam assisted deposition. Nucl. Instrum. Methods Phys. Res. Sect. B.

[30] Inerbaev TM, Sahara R, Mizuseki H, Kawazoe Y, Nakamura T (2007). Interstitial oxygen and dopant atoms arrangement in tin-doped indium oxide. Mater. Trans..

[31] Hou Q, Buckeridge J, Lazauskas T, Mora-Fonz D, Sokol AA, Woodley SM, Catlow CRA (2018). Defect formation in In_2_O_3_ and SnO_2_: A new atomistic approach based on accurate lattice energies. J. Mater. Chem. C.

[32] Buckeridge J, Catlow CRA, Walsh A (2018). Deep vs shallow nature of oxygen vacancies and consequent n-type carrier concentrations in transparent conducting oxides. Phys. Rev. Mater..

[33] Vasudev AP, Kang JH, Park J, Liu X, Brongersma ML (2013). Electro-optical modulation of a silicon waveguide with an “epsilon-near-zero” material. Opt. Express.

[34] Rajput S, Kaushik V, Kumar M (2021). Optical modulation via coupling of distributed semiconductor heterojunctions in a Si-ITO-based subwavelength grating. Phys. Rev. Appl..

[35] Kühnle J, Bergmann RB, Krinke J, Werner JH (1996). Comparison of vapor phase and liquid phase epitaxy for deposition of crystalline Si on glass. MRS Online Proc. Libr. OPL..

[36] Li H, Guo L, Chen Y (2020). High temperature conductive stability of indium tin oxide films. Front. Mater..

[37] Amin R, Maiti R, George JK (2020). A lateral MOS-capacitor-enabled ITO Mach–Zehnder modulator for beam steering. J. Lightwave Technol..

[38] Baburin, A. S., Ivanov, A. I., Ryzhikov, I. A. *et al.* Crystalline structure dependence on optical properties of silver thin film over time. In* 2017 Progress in Electromagnetics Research Symposium-Spring (PIERS)*, 1497–1502. (IEEE, 2017). 10.1109/PIERS.2017.8261984.

[39] Rodionov IA, Baburin AS, Rizhikov IA (2017). Mass production compatible fabrication techniques of single-crystalline silver metamaterials and plasmonics devices. Metamater. Metadevices Metasyst..

[40] Forouzmand A, Mosallaei H (2019). Electro-optical amplitude and phase modulators based on tunable guided-mode resonance effect. ACS Photonics.

[41] Quaas M, Steffen H, Hippler R, Wulff H (2003). Investigation of diffusion and crystallization processes in thin ITO films by temperature and time resolved grazing incidence X-ray diffractometry. Surf. Sci..

[42] Rodionov IA, Baburin AS, Gabidullin AR (2019). Quantum engineering of atomically smooth single-crystalline silver films. Sci. Rep..

