# Supplementary material for: ITO film stack engineering for low-loss silicon optical modulators
Source: Sci Rep. 2022 Apr 15;12:6321. doi: 10.1038/s41598-022-09973-5 (PMC9012746; doi:10.1038/s41598-022-09973-5)
Supplement: Supplementary file 1 — Supplementary Information. [file 41598_2022_9973_MOESM1_ESM.docx]

**Supplementary Information**

**ITO Film Stack Engineering for Low-Loss Silicon Optical Modulators**

*Evgeniy S. Lotkov^1,2,*^, Alexander S. Baburin^1,2^, Ilya A. Ryzhikov^1,3^, Olga S. Sorokina^1,2^, Anton I. Ivanov^1,2^, Alexander V. Zverev^1,2^, Vitaly V. Ryzhkov^1^, Igor V. Bykov^3^, Alexander V. Baryshev^2^, Yuri V. Panfilov^1^, and Ilya A. Rodionov^1,2^*

^1^FMN Laboratory, Bauman Moscow State Technical University, Moscow, Russia

^2^Dukhov Automatics Research Institute, (VNIIA), Moscow, Russia

^3^Institute for Theoretical and Applied Electromagnetics RAS, Moscow, Russia

*e-mail: lotevg@bmstu.ru

**Table of content**

1. **Carrier concentration analyze**
2. **Detailed description of the deposited ITO films electrical parameters**
3. **Details of the electro-optical modulation experiments**
4. **The device bandwidth analyze**
5. **Supplementary Information Figures**
6. **Carrier concentration analyze**

It is necessary to select the optimal carrier concentration in the ITO film, that is, the concentration at which the device will have the lowest light propagation loss (IL) and the minimize length of the active part (in which modulation occurs). In the case of using the design of the Mach-Zehnder interferometer, the device active part length (L) should be such that a complete phase shift of the light (*Δφ = π*) in one of the arms (1) is provided.

| $\Delta\varphi=\frac{2\pi}{\lambda}L\Delta n_{eff}$ | (1) |
| --- | --- |

The change in the effective refractive index *Δn_eff_* is determined by a few factors: the refractive index change in ITO (*Δn_ITO_*), the bias in the MOS capacitor (*V_g_*), and the design of the device (the thickness of the ITO and oxide films, the cross section of the waveguide, the location of the electrode, etc.).

ITO index change *Δn_ITO_* depends on the initial carrier concentration of the ITO film *N_c_* [1]. We analyzed ITO films with a number of values of the carrier concentrations *N_c_* (1·10^19^…4·10^20^ cm^-3^) using the Drude-Lorentz model, taking $\mu_{c}=20\frac{{cm}^{2}}{V\cdot s}$ (equations 2).

| $\varepsilon_{r}=\varepsilon_{\infty}-\frac{\omega_{p}^{2}}{\omega^{2}+\gamma^{2}}+i\frac{\omega_{p}^{2}\gamma}{\left( \omega^{2}+\gamma^{2} \right)\omega}; \omega_{p}^{2}=\frac{N_{c}e^{2}}{\varepsilon_{0}m^{*}}; \gamma=\frac{e}{\mu_{c}m^{*}}$ | (2) |
| --- | --- |

The dependence of the spectra of the refractive index *n_ITO_* and the absorption coefficient *k_ITO_* on the carrier concentration is shown in Fig. S1.

The change in the carrier concentration under the conditions of charge accumulation / depletion in an ITO film at an applied voltage *V_g_* can be calculated using equation (3).

| $\Delta N_{c}=\frac{\varepsilon_{0}\varepsilon_{ITO}V_{g}}{t_{SiO2}t_{acc}e} ,$ | (3) |
| --- | --- |

where *ε_0_* is the dielectric constant of vacuum, *ε_ITO_* is the relative permittivity of ITO, *e* is the electron charge, *t_SiO2_* is the oxide thickness, *t_acc_* is the thickness of the accumulating layer, which can be calculated from the Thomas-Fermi shielding theory (4) [5].

| $t_{acc}=\left[ \frac{\varepsilon_{ITO}\varepsilon_{0}h^{2}}{4\pi^{2}m_{eff}e^{2}} \right]^{\frac{1}{2}}\left[ \frac{\pi^{4}}{3N_{0}} \right]^{\frac{1}{6}}$ | (4) |
| --- | --- |

where *N_0_* is the initial concentration of charge carriers in the ITO film, *m_eff_* is an effective electron mass.

Thus, it is possible to calculate the value of the ITO carrier concentration under conditions of accumulation *N_c_^+^ = N_c_ + ΔN_c_* and under conditions of depletion *N_c_^–^ = N_c_ – ΔN_c_*. Knowing these values, using the Drude-Lorenz model, it is possible to find the refractive index and absorption coefficient under the conditions of accumulation *n^+^*, *k^+^* and depletion *n–*, *k–*. To analyze the parameters of the device, operating voltages of ± 6, ± 13, ± 16 V were selected.

The characteristics *Δn_eff_ = n_eff_^–^ – n_eff_^+^* and *Δk_eff_ = k_eff_^+^ – k_eff_^–^* can be derived by substituting the values of *n^+^*, *k^+^* and *n^–^*, *k^–^* into the COMSOL model with a typical design [2], which is shown in Fig. S2.

It is possible to find the propagation loss per unit length in the active part of the device using the equation (5). For the correct calculation of the modulator insertion loss *IL*, it is necessary to choose the maximum value of the extinction coefficient k that the Mach-Zehnder modulator can have in one arm. Despite the fact that the Mach-Zehnder design controls the change in refractive index (in one arm), the absorption coefficient also undergoes changes due to the properties of the ITO.

| $\alpha[dB/um]=\frac{2\pi k_{eff}^{+}10{log}_{10} (e)}{\lambda[um]}=\frac{2\pi k_{eff}^{+}4.34}{\lambda[um]}$ | (5) |
| --- | --- |

Having determined the length of the active part of the device *L* from relation (1), we find the propagation loss *IL* over the entire length (6). It should be noted that, we cannot analyze the total on-chip losses and restrict ourselves only to calculating the losses inside the active element (coupling losses to and from the Si waveguide were not taken into account).

| $IL[dB]=\alpha[dB/um]\cdot L[um]$ | (6) |
| --- | --- |

The dependence of the device length and propagation loss on the initial carrier concentration of the ITO film is shown in Fig. S3. The device length has the smallest value at the point N_c_ = 5.5·10^20^ cm^-3^ where ITO has a peak (maximum) of the refractive index change *Δn_ITO_* (this is epsilon-near-zero point of our model) but the propagation loss in this point are colossal (> 30 dB which is equivalent to almost complete loss of light in the waveguide). With increased operating voltage of the device, the light propagation loss IL decreases. With increased operating voltage of the device, the light propagation loss IL decreases. There are at least 2 reasons which leads to advantages with the voltage increasing: 1) it strongly affects the device length leading to IL decrease; 2) affect *ΔN_c_* increase leading to increase of $k_{eff}^{+}, \alpha$ and *IL*. The second one is not such a strong effect compared to the first one.

The low carrier concentration of the ITO film will lead to a longer charging time in the modulator capacitor. However, given the small thickness (10-20 nm) and the film footprint in the device (approximately 50 μm^2^), it is possible to neglect the low ITO conductivity without losing high bandwidth (see section «The device bandwidth analyze»). On the other side, the low ITO conductivity will provide a significant reduction in optical losses in the Mach-Zehnder design [1].

Thus, there is a trade-off between device length and propagation loss. The optimal range of carrier concentration for ITO film is from 0.5·10^20^ to 2·10^20^ cm^-3^ where the values of the device parameters find a trade-off.

1. **Detailed description of the deposited ITO films electrical parameters**

Subsequent annealing of room temperature films evaporated without ion beam assistance dramatically lower the resistivity to 2.5·10^-4^ Ω·cm (Fig. S4a, type4). Ion-beam assistance with Ar/O_2_ mixture and elevated temperature during evaporation (without annealing) leads to ITO films with a higher resistance value of 15.8·10^-4^ Ω·cm (Fig. S4a, type1). Subsequent annealing of high temperature IBAD ITO films distinguishably lower resistance from 15.8·10^-4^ to 4·10^-4^ Ω·cm (Fig. S4a, type3). Finally, IBAD ITO films evaporated at room temperature with subsequent annealing leads to the medium value of resistivity around 11·10^‑4^ Ω·cm (Fig. S4a, type2).

The psi delta dependences in the wavelength range from 400 to 1600 nm and the Drude-Lorentz parameters for ITO films 1-3 are shown in Fig. S4d (blue line – measured curve, red dashed line – fitted curve). We used ellipsometry model ITO/Si without native oxide because the films was deposited on the Si substrate cleaned in HF (see Methods). We did not consider the index gradient [3, 4] because we had a good fit to the measured data (MSE < 0.5). Carrier concentrations for ITO film types 1-3 are calculated based on the measured plasma frequency ω_p_ by ellipsometry (Table S1).

For all other obtained films in the work (deposition and annealed experiments of film type2), we only used 4-probe resistivity measurements to calculate the approximate carrier concentration values and tracked their correlation with the Tauc-Lorentz model. We calculated the carrier concentrations from the resistivity values (Fig. S5a). The carrier mobility was estimated as 10 cm^2^/(V·s) for as deposited films and 15 cm^2^/(V·s) for annealed films. To correlate the results with the optical performance of the films, we calculated the extinction coefficient rise wavelength value in the UV range for each ITO film, using the Tauc-Lorentz model (Fig. S5b). This wavelenght must shift further into UV range at rising of the film carrier concentration. For annealed films (Fig. S5d), the tendency of the carrier concentration values and the extinction coefficient rise wavelength values almost matches. However, for as deposited films, the tendency is quite violated, which indicates a discrepancy of the carrier mobility in the points Ar/O_2_ flow = 4/8, 2/12. This can be caused both by a violation of the stoichiometry and by the imperfection of optical measurements in the UV range for as deposited films.

The same sequence was performed for resistivity values in the annealed experiments (Fig. 2a from the main text). The tendency of the carrier concentration values (Fig. S6a) and the extinction coefficient rise wavelength values (Fig. S6b) also completely matches (Fig. S6c).

1. **Details of the electro-optical modulation experiments**

In all calculations of Δn, Δk, we used the Drude-Lorenz model in the range of 550-1600 nm. The sequence for measuring and calculating the data was as follows:

- Measurement of psi, delta at 0 V and calculation of Drude parameters (one ITO layer in model), minimizing MSE (0.11-0.58 for different films);

- Measurement of psi, delta at (+ some V);

- Dividing ITO into 2 layers in the model (1 – a layer with the same parameters as the layer at 0V, all parameters are fixed; 2 – an accumulation layer, the thickness of which was approximately estimated from the Thomas-Fermi equation (7) [5]). The resulting accumulation layer thickness was found to be between 0.7 and 1 nm (for different ITO films carrier concentration). The Drude parameters of the accumulation layer are calculated in the next step with specification of thickness;

| $\lambda_{TF}=\left[ \frac{\varepsilon_{ITO}\varepsilon_{0}h^{2}}{4\pi^{2}m_{eff}e^{2}} \right]^{\frac{1}{2}}\left[ \frac{\pi^{4}}{3N_{0}} \right]^{\frac{1}{6}},$ | (7) |
| --- | --- |

- Change the thickness of the accumulation layer until the MSE is minimal (0.11-0.34 for different films). The value of the found thickness differs upwards from the calculated one (from +0.2 to +1 nm for films with different concentrations, it is caused because of accumulation layer represents the carrier concentration gradient and not defined by only one layer).

This simplified approach showed good agreement between the results and the carrier concentration values.

See the fitted Drude parameters for each film in the Tables S2-S4.

Film type1 has a very low psi-delta change as well as poor goodness of fit in the ITO / SiO_2_ two-layer coating. Therefore, for the most sensitive calculation, the Drude-Lorentz oscillator was improved to three oscillators (to minimize the error). The calculated parameters of this film are shown in the Table S2.

ITO film type3 shows maximum shift in the range from +16 V to -16 V (since it is the closest to ENZ point) – Δn = 0.199, Δk = 0.240 (Table S5). However, it has the highest extinction coefficient in the IR range that will result in higher IL of an electro-optical modulator. ITO film type2 is the most suitable for an electro-optical modulator since it has the lowest extinction coefficient in the IR range and is comparable to film type3 in terms of electro optical effect (Δn = 0.101, Δk = 0.084) in the voltage range from –16 V to +16 V.

1. **The device bandwidth analyze**

We consider the plasmonic design with the p-type doped silicon waveguide as a bottom electrode and ITO as a top electrode (Fig. S7). The main disadvantages of such solution are the bandwidth limitation and additionally increased IL due to the Si contact to the waveguide placed outside of the device region. Nevertheless, this design is preferred for our future work, since thermal oxide could be used as a reliable dielectric layer [6] and it has the simplified fabrication technology (compared to the devices that require the oxide window etching operations to provide contact to the ITO layer [2]). These technology features allow us to develop low-loss EOM with low switching voltage, which is an advantage for silicon photonics devices.

There are two schemes (Fig. S7): lateral (without electrode on top) and vertical (with electrode on top). To optimize the design we chose the next geometry parameters:

*L_1_* = 5 um (see Fig. S3 for V_π_ = ±16 V and N_c_ = 8·10^19^ cm^-3^ – the minimum carrier concentration obtained in our work),

*L_2_* = 2 um, *L_3_* = 2 um, *W* = 0.5 um, *W_1min_* = 1 um, *W_c_* = 0.3 um.

The resistivity of ITO was taken as 5.4·10^-5^ Ω·m (correspond to N_c_ = 8·10^19^ cm^-3^) and resistivity of the silicon waveguide was 5·10^-5^ Ω·m (corresponding to the light-doped p-Si).

The active element resistance:

$$R_{Si}=\rho_{Si}\left( \frac{L_{1}+L_{2}}{Wd_{Si}}+\frac{L_{3}}{W_{с}d_{Si}} \right)=4700 \Omega;$$

$$R_{ITO1}=\rho_{ITO}\frac{W+W_{1min}}{{L_{1}d}_{ITO}}=2058 \Omega;$$

$$R_{ITO2}=\rho_{ITO}\frac{d_{ITO}}{WL_{1}}=1.1 \Omega;$$

where *R_Si_* – resistivity of the Si waveguide, *R_ITO1_* – ITO layer resistivity in the lateral electrode scheme, *R_ITO2_* – ITO layer resistivity in the vertical electrode scheme, *d_ITO_* – ITO thickness, *d_Si_* – Si waveguide thickness.

Taking into account a parasitic resistance *R_p_* between the metal electrode and the Si or ITO area (3.5 kΩ is taken as in the work [7]), the total resistivity of the modulator conductive elements will be as follow:

$$Lateral electrode: R=R_{Si}+R_{ITO1}+{2R}_{p}=13.8 k\Omega;$$

$$Vertical electrode: R=R_{Si}+R_{ITO2}+{2R}_{p}=11.7 k\Omega;$$

The active element capacity:

$$C=\varepsilon_{0}\varepsilon_{r}\frac{L_{1}W}{d_{oxide}}=4 fF;$$

where *ε_r_* = 3.9 – SiO_2_ relative static permittivity, *d_oxide_* – oxide thickness.

The device theoretical 3dB frequency:

$$Lateral electrode: f_{3dB}=\frac{1}{2\pi RC}=2.9 GHz;$$

$$Vertical electrode: f_{3dB}=\frac{1}{2\pi RC}=3.4 GHz.$$

Thus, the 5 um – length device has the bandwidth of 3.4 GHz in the case of the vertical electrode and 2.9 GHz in the case of the lateral electrode scheme.

It should be noted that in this work we were only interested in the estimation of the bandwidth and insertion loss, but not the extinction ratio, which can be estimated only after the device has been fabricated (since this parameter is determined not by the active element in the Mach-Zehnder design, but mainly by the coupler located at the end of the phase-shifter). The optical modulator parameters have been optimized only by ITO carrier concentration in this work. Optimizing the design using multi-layer MOS [8], multi-slot waveguide [9], etc. the devise with the much lower propagation losses (<1 dB) and high bandwidth (> 25 GHz) can be obtained.

1. **Supplementary Information Figures**


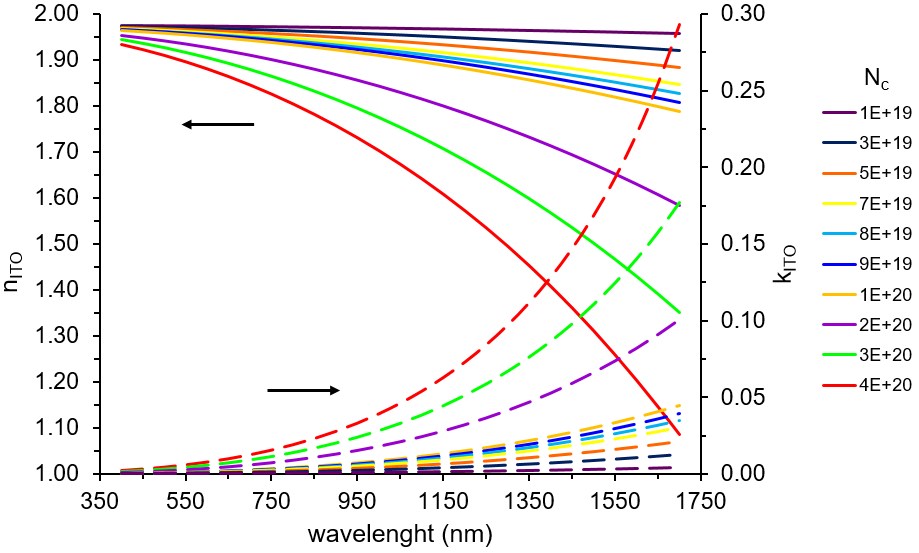


**Fig. S1** – Dependence of the *n_ITO_* and *k_ITO_* spectra on the carrier concentration of the ITO film.


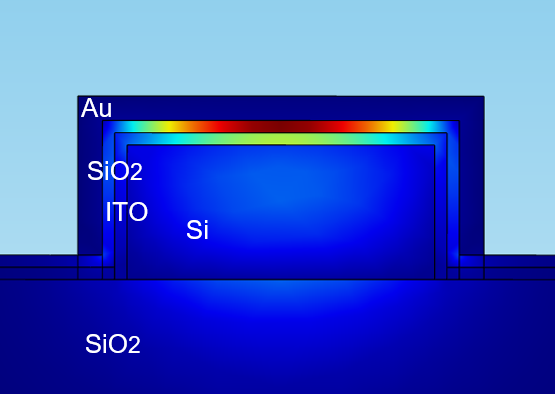


**Fig. S2** – COMSOL simulation for extracting *Δn_eff_* and *Δk_eff_* values.


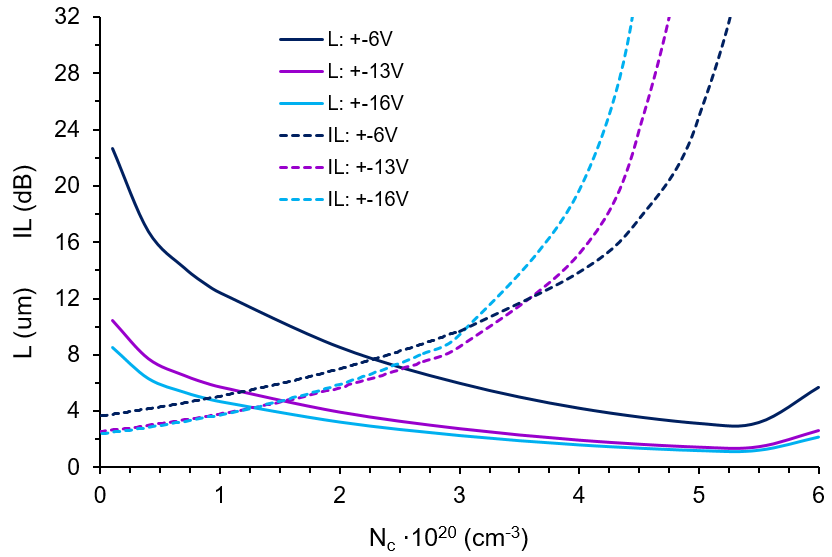


**Fig. S3** – Dependence of device parameters on the concentration of ITO film carriers.

| 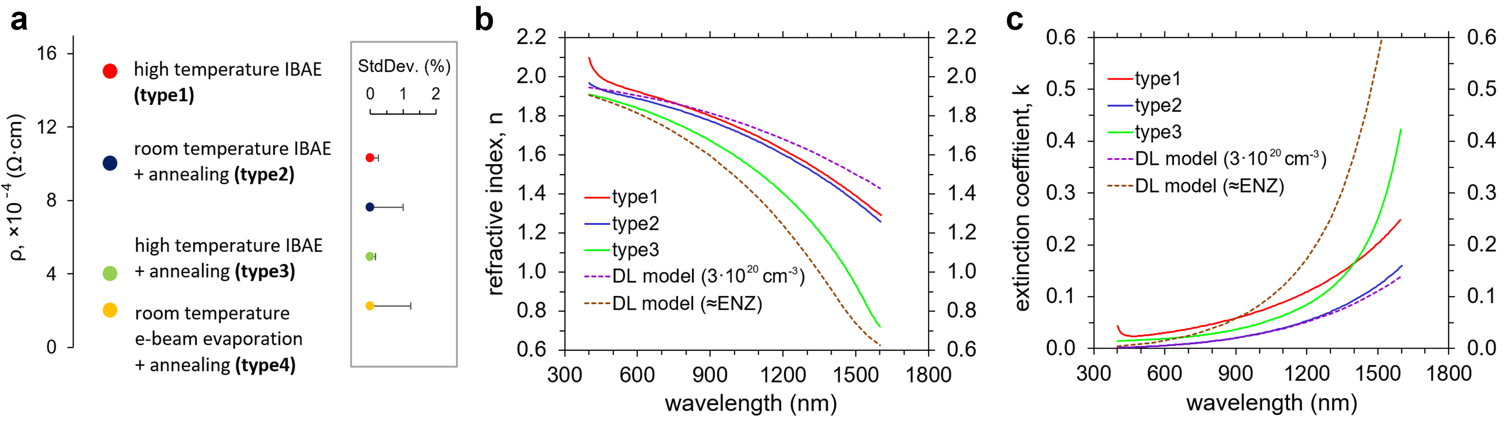  **d/** | | |
| --- | --- | --- |
| **film 1 (MSE = 0.507)** | **film 2 (MSE = 0.295)** | **film 3 (MSE = 0.457)** |
| 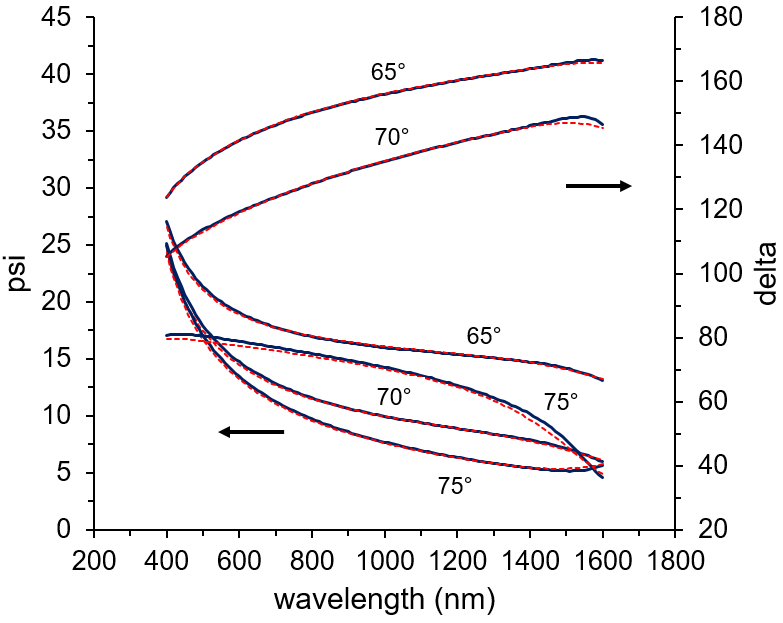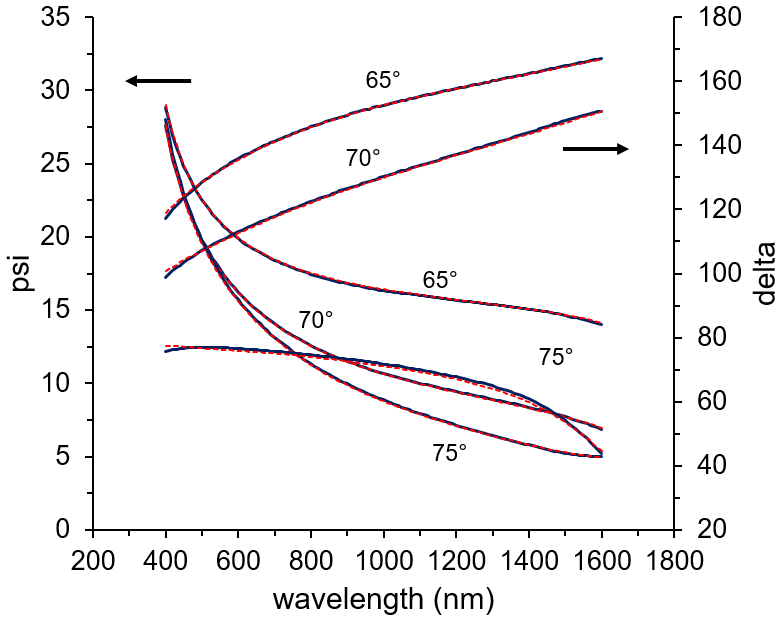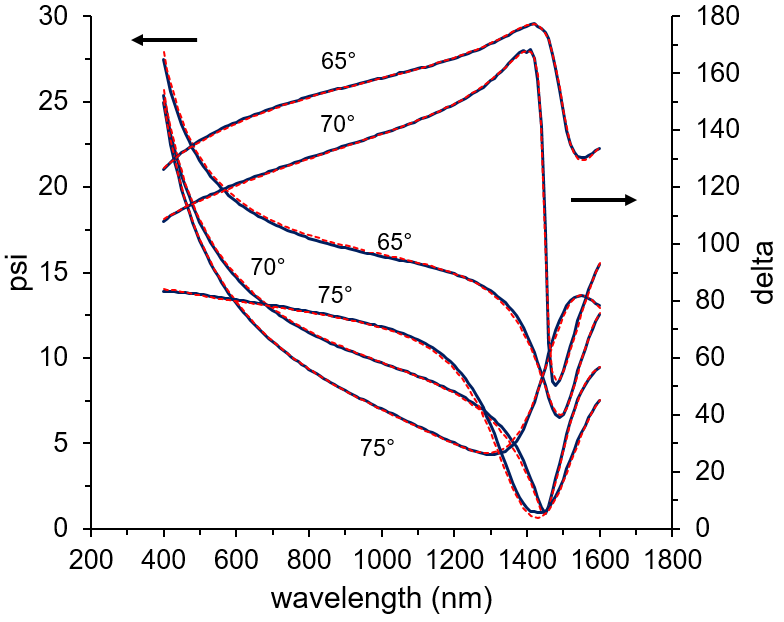 | | |
| Drude parameters:  ε_inf real_ = 3.9  ε_inf imag_ = 0.025  ω_p_ = 10389 cm^-1^  γ = 1831 cm^-1^ | Drude parameters:  ε_inf real_ = 3.9  ε_inf imag_ = 0.000  ω_p_ = 9693 cm^-1^  γ = 1090 cm^-1^ | Drude parameters:  ε_inf real_ = 3.9  ε_inf imag_ = 0.005  ω_p_ = 12160 cm^-1^  γ = 890 cm^-1^ |

**Fig. S4** – a) ITO films resistivity via deposition technique; ITO films b) n and c) k dependence on wavelength; d) ITO films ellipsometry fitting parameters.

| 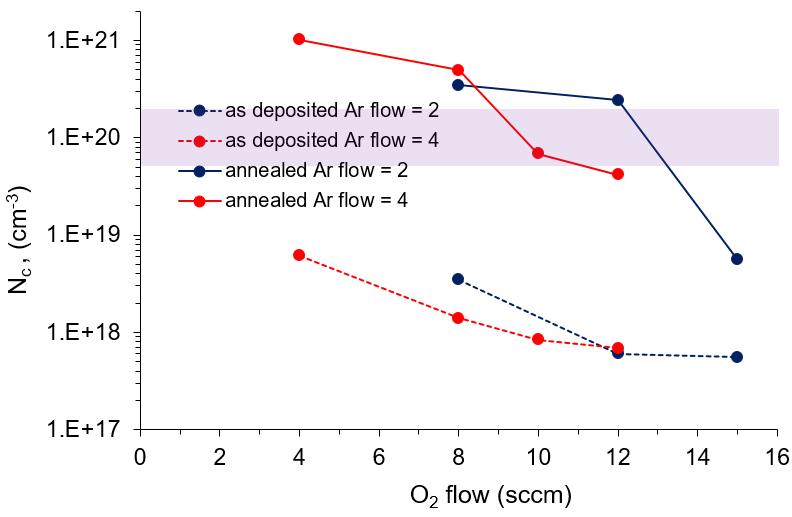  **a/** | |
| --- | --- |
| 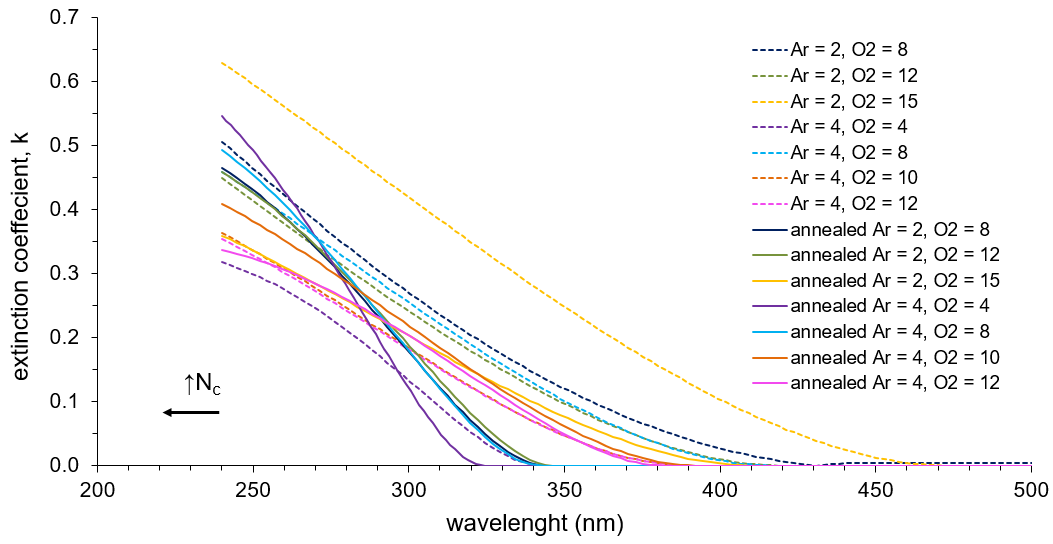  **d/**  **c/**  **b/** |  |
| 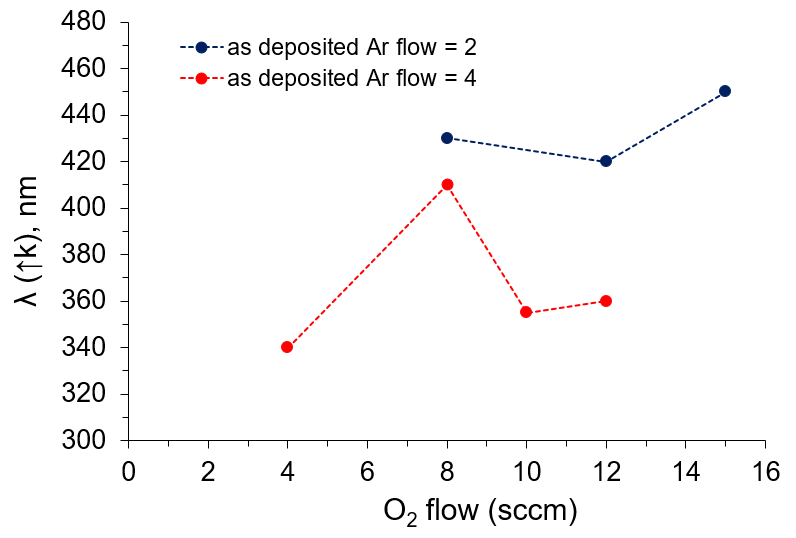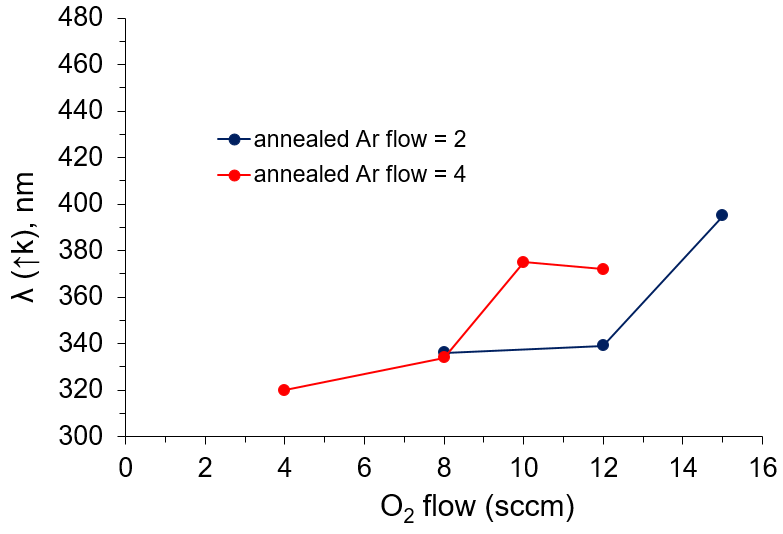 |  |

**Fig. S5** – Carrier concentration correlating for deposition experiments: a) N_c_ dependence on IBAE parameters; b) ITO films extinction coefficient in the UV range; c) as deposited and d) annealed ITO films ***κ*** edge analysis

**a**

| 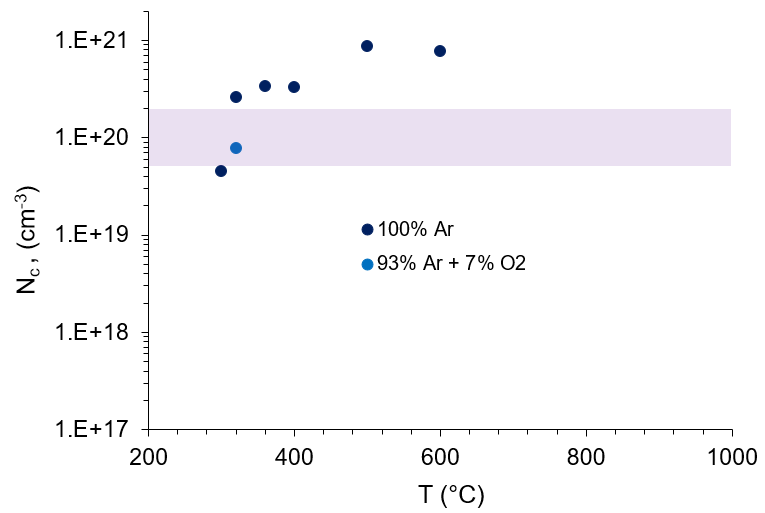  **b**  **c** | |
| --- | --- |
| 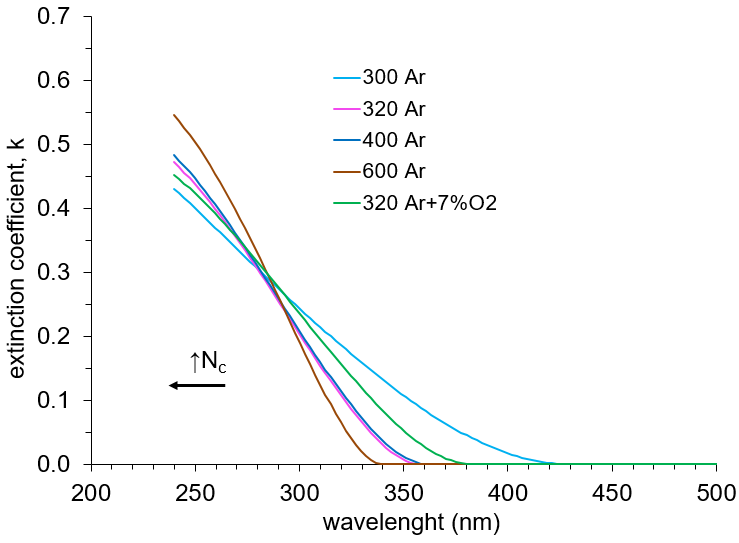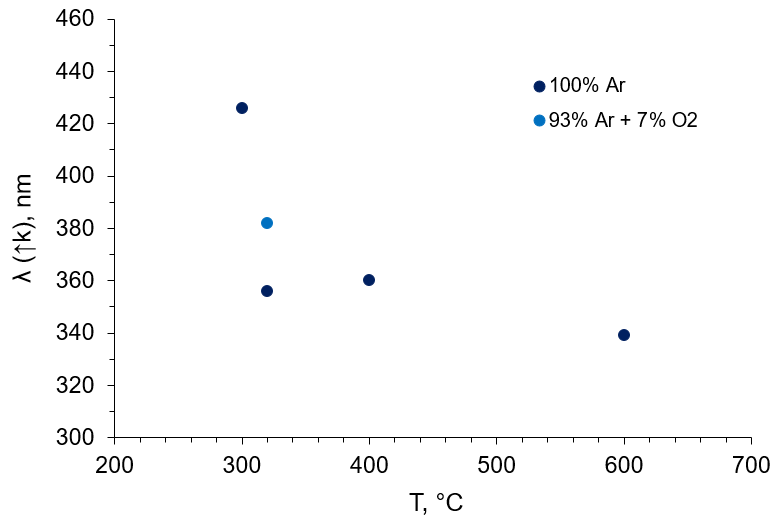 |  |

**Fig. S6** – Carrier concentration correlating for annealing experiments: a) N_c_ dependence on annealing temperature and atmosphere; b) ITO films extinction coefficient in the UV range; c) ***κ*** edge analysis for the annealed ITO films

| 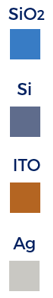 | 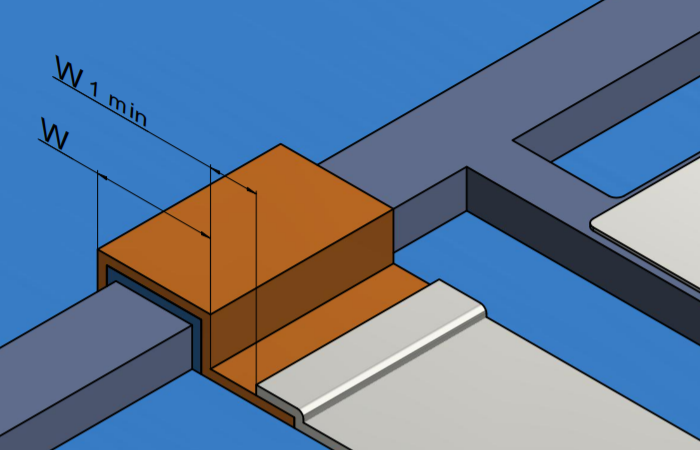 | 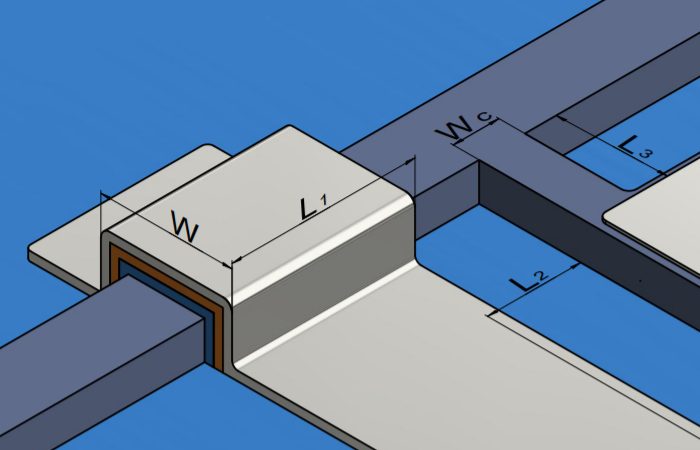 |
| --- | --- | --- |
|  | 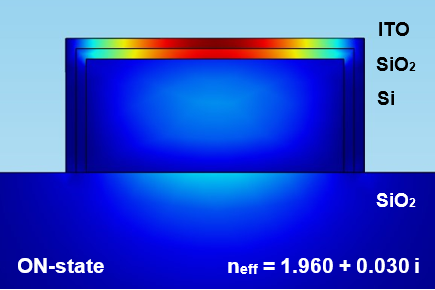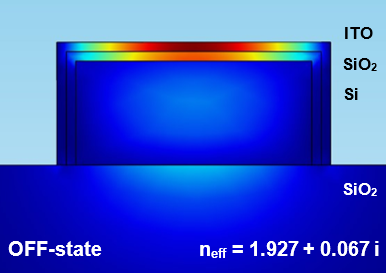 | 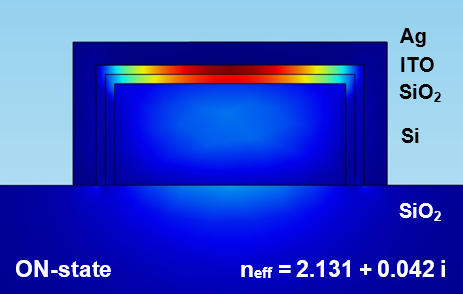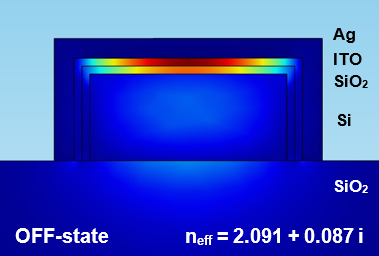 |

**Fig. S7** – Comparison of lateral (left) electrode scheme and vertical (right) electrode scheme, W – MOS-capacitor width, W_1 min_ – minimum distance to the electrode to eliminate the transient charge distribution, W_c_ – Si contact path width, L_1_ – MOS-capacitor length, L_2_ – distance between two electrodes, L_3_ – Si contact path length.

**Table S1**. Electrical parameters of ITO film types 1-3.

| **Deposition technique** | **Plasma frequency ω_p_, cm^-1^** | **N_c_, cm^-3^** | **ρ (measured), Ω·cm** | **μ_c_, cm^2^/(V·s)** |
| --- | --- | --- | --- | --- |
| type1 | 10389 | 4.2·10^20^ | 1.58·10^-3^ | 9.4 |
| type2 | 9693 | 3.7·10^20^ | 1.1·10^-3^ | 15.5 |
| type3 | 12160 | 5.7·10^20^ | 4.0·10^-4^ | 27.0 |

**Table S2.** Drude parameters for film type1 (fitted accumulation layer thickness is 1.51 nm);

ΔN_c_ = 3.1·10^19^ cm^-3^.

|  | 0V | +16V | -16V |
| --- | --- | --- | --- |
| MSE | 0.34 | 0.31 | 0.34 |
| Epsilon-infinity-real | 3.78 | 3.78 | 3.78 |
| Epsilon-infinity-imag | 0.001 | 0.001 | 0.001 |
| w-p-free-carriers-(1/cm) | 10282.71 | 10458.29 | 10067.27 |
| w-tau-free-carriers-(1/cm) | 1556.89 | 1489.81 | 1129.41 |
| (1)Omega-O-(1/cm) | 79.86 | 69.54 | 141.65 |
| (1)Omega-p-(1/cm) | 6562.81 | 6549.83 | 6477.78 |
| (1)Omega-tau-(1/cm) | 1463.28 | 1478.50 | 1441.00 |
| (2)Omega-O-(1/cm) | 39949.92 | 32444.58 | 38768.79 |
| (2)Omega-p-(1/cm) | 7574.95 | 7093.67 | 6750.20 |
| (2)Omega-tau-(1/cm) | 0.07 | 0.47 | 0.06 |
| (3)Omega-O-(1/cm) | 13219.25 | 14734.51 | 13249.77 |
| (3)Omega-p-(1/cm) | 1252.15 | 2941.85 | 1272.78 |
| (3)Omega-tau-(1/cm) | 1675.73 | 917.26 | 1740.14 |

**Table S3.** Drude parameters for film type2 (fitted accumulation layer thickness is 1.79 nm);

ΔN_c_ = 9.1·10^19^ cm^-3^.

|  | 0V | +16V | -16V |
| --- | --- | --- | --- |
| MSE | 0.11 | 0.11 | 0.12 |
| w-p-free-carriers-(1/cm) | 9711 | 10393 | 9212 |
| w-tau-free-carriers-(1/cm) | 1125 | 1374 | 854 |

**Table S4.** Drude parameters for film type3 (fitted accumulation layer thickness is 0.98 nm);

ΔN_c_ = 2.0·10^20^ cm^-3^.

|  | 0V | +16V | -16V |
| --- | --- | --- | --- |
| MSE | 0.29 | 0.27 | 0.27 |
| w-p-free-carriers-(1/cm) | 12014 | 12772 | 11431 |
| w-tau-free-carriers-(1/cm) | 776 | 924 | 457 |

**Table S5.** Comparison of refractive index and extinction coefficient change for ITO film types 1-3 at λ = 1550 nm.

| **Parameter** | **type1** | **type2** | **type3** |
| --- | --- | --- | --- |
| **N_c_ , cm^-3^** | **4.2·10^20^** | **3.7·10^20^** | **5.7·10^20^** |
| Δn (0 + 16 V) | 0.053 | 0.048 | 0.101 |
| Δn (0 - 16 V) | 0.030 | 0.053 | 0.098 |
| Δk (0 + 16 V) | 0.030 | 0.028 | 0.119 |
| Δk (0 - 16 V) | 0.016 | 0.056 | 0.121 |
| **Δn** | **0.083** | **0.101** | **0.199** |
| **Δk** | **0.046** | **0.084** | **0.240** |
| n (at 0V) | 1.342 | 1.308 | 0.675 |
| k (at 0V) | 0.226 | 0.141 | 0.329 |

**Supplementary Information References**

1. Amin, R., Suer, C., Ma, Z., Sarpkaya, I., Khurgin, J. B., Agarwal, R., & Sorger, V. J. Active material, optical mode and cavity impact on nanoscale electro-optic modulation performance. Nanophotonics **7**, 455-472; https://doi.org/10.1515/nanoph-2017-0072 (2017).

2. Amin, R., Maiti, R., Gui, Y. et al. Heterogeneously integrated ITO plasmonic Mach–Zehnder interferometric modulator on SOI. Sci Rep **11**, 1287; https://doi.org/10.1038/s41598-020-80381-3 (2021).

3. Sytchkova, A., Zola, D., Bailey, L. R., Mackenzie, B., Proudfoot, G., Tian, M., & Ulyashin, A. Depth dependent properties of ITO thin films grown by pulsed DC sputtering. Materials Science and Engineering: B **178**, 586-592; https://doi.org/10.1016/j.mseb.2012.11.010 (2013).

4. Gui, Y., Miscuglio, M., Ma, Z. et al. Towards integrated metatronics: a holistic approach on precise optical and electrical properties of Indium Tin Oxide. Sci Rep **9**, 11279; https://doi.org/10.1038/s41598-019-47631-5 (2019).

5. Melikyan, A., Lindenmann, N., Leuthold, J., et al. Surface plasmon polariton absorption modulator. Optics express **19**, 8855-8869 (2011).

6. J. H. Stathis. Reliability limits for the gate insulator in CMOS technology. IBM Journal of Research and Development, 46, 265-286; https://doi.org/10.1147/rd.462.0265 (2002).

7. Zan et al. A study of parasitic resistance effects in thin-channel polycrystalline silicon TFTs with tungsten-clad source/drain. IEEE Electron Device Letters **24**; https://doi.org/10.1109/LED.2003.815160 (2003).

8. Qiu, X., Ruan, X., Li, Y., & Zhang, F. Multi-layer MOS capacitor based polarization insensitive electro-optic intensity modulator. Optics express **26**, 13902-13914; https://doi.org/10.1364/OE.26.013902 (2018).

9. Sha, Y., Wu, J., Xie, Z. T., Fu, H. Y., & Li, Q. Comparison study of multi-slot designs in epsilon-near-zero waveguide-based electro-optical modulators. IEEE Photonics Journal **13**, 1-12; https://doi.org/10.1109/JPHOT.2021.3084943 (2021).
